# Spermatid-specific linker histone HILS1 is a poor condenser of DNA and chromatin and preferentially associates with LINE-1 elements

**DOI:** 10.1186/s13072-018-0214-0

**Published:** 2018-08-01

**Authors:** Laxmi Narayan Mishra, Vasantha Shalini, Nikhil Gupta, Krittika Ghosh, Neeraj Suthar, Utsa Bhaduri, M. R. Satyanarayana Rao

**Affiliations:** 1 Chromatin Biology Laboratory, Molecular Biology and Genetics Unit, Jawaharlal Nehru Centre for Advanced Scientific Research, Jakkur P.O., Bangalore, 560064 India; 2 Present Address: Department of Biochemistry and Biophysics, University of Rochester Medical Center, Rochester, NY 14642 USA; 3 Present Address: Epigenetics and Cell Fate, UMR7216, CNRS, University Paris Diderot, Sorbonne Paris Cite, 75013 Paris, France; 4 InterpretOmics India Pvt. Ltd., #329, 7th Main, HAL II Stage 80 Feet Road, Indira Nagar, Bangalore, 560008 India

**Keywords:** Spermiogenesis, Chromatin remodeling, Linker histone, Circular dichroism, ChIP sequencing

## Abstract

**Background:**

Linker histones establish and maintain higher-order chromatin structure. Eleven linker histone subtypes have been reported in mammals. HILS1 is a spermatid-specific linker histone, and its expression overlaps with the histone–protamine exchange process during mammalian spermiogenesis. However, the role of HILS1 in spermatid chromatin remodeling is largely unknown.

**Results:**

In this study, we demonstrate using circular dichroism spectroscopy that HILS1 is a poor condenser of DNA and chromatin compared to somatic linker histone H1d. Genome-wide occupancy study in elongating/condensing spermatids revealed the preferential binding of HILS1 to the LINE-1 (L1) elements within the intergenic and intronic regions of rat spermatid genome. We observed specific enrichment of the histone PTMs like H3K9me3, H4K20me3 and H4 acetylation marks (H4K5ac and H4K12ac) in the HILS1-bound chromatin complex, whereas H3K4me3 and H3K27me3 marks were absent.

**Conclusions:**

HILS1 possesses significantly lower α-helicity compared to other linker histones such as H1t and H1d. Interestingly, in contrast to the somatic histone variant H1d, HILS1 is a poor condenser of chromatin which demonstrate the idea that this particular linker histone variant may have distinct role in histone to protamine replacement. Based on HILS1 ChIP-seq analysis of elongating/condensing spermatids, we speculate that HILS1 may provide a platform for the structural transitions and forms the higher-order chromatin structures encompassing LINE-1 elements during spermiogenesis.

**Electronic supplementary material:**

The online version of this article (10.1186/s13072-018-0214-0) contains supplementary material, which is available to authorized users.

## Background

The nucleosome is the basic unit of chromatin, comprised of 146 bp of DNA wrapped around an octamer of core histones [1]. Nucleosomes are connected to each other with a segment of DNA known as linker DNA. Linker histones are lysine-rich proteins associated with the DNA entering and exiting the nucleosomes sealing two turns of DNA around the core histone octamer. Linker histone binding to the nucleosome protects extra 20 bp of DNA and the structure is called chromatosome. Linker histones help in the formation and stabilization of 30 nm fiber [2] and facilitate the self-association of fibers into oligomeric tertiary chromatin structures [3]. In mammals, eleven linker histone variants have been reported, of which, seven are somatic subtypes like H1a (H1.1), H1b (H1.5), H1c (H1.2), H1d (H1.3), H1e (H1.4), H1x (H1.10), and H1^0^ (H1.0); three are testis-specific like H1t (H1.6), H1T2 (H1.7), and HILS1 (H1.9); and H1oo (H1.8), which is an oocyte-specific linker histone [4–6]. H1 variants differ from each other in their ability to condense DNA and chromatin in vitro [7, 8] and also exhibit different chromatin binding affinity [9]. Linker histones have a three-dimensional tripartite structure that includes a short N-terminal basic domain (NTD); a conserved and hydrophobic middle globular domain (GD); and a highly basic C-terminal domain (CTD) [10]. Both NTD and CTD lack defined structures in aqueous solution but attain secondary structures in the presence of macromolecules such as DNA and thus linker histones are intrinsically disordered [2, 3, 11]. The globular domain of linker histones possesses two regions of DNA binding sites that facilitate chromatosome formation [3–6, 12–15]. The GD directs the CTD to the nucleosome, facilitating the formation of the chromatosome particle [16–19]. The CTD of linker histones facilitates and stabilizes chromatin folding [19–22]. Most of the known linker histones have S/TPXK motifs in the CTD that are responsible for chromatin condensation in vitro [7–13, 23, 24]. To elucidate the unique functions of linker histones, knockout mice have been created. Elimination of single H1 subtype, H1^0^, H1a, H1c, H1d, H1e, and H1t or double H1 knockout (H1.0/H1c, H1.0/H1d, or H1.0/H1e) does not perturb mouse development, and they maintain a normal H1-to-nucleosome stoichiometry by upregulation of remaining subtypes [25–29]. However, triple-H1-null (H1c, H1d, and H1e) mouse embryos have ~ 50% of the normal H1-to-nucleosome ratio and they die by mid-gestation [30]. Depending on the linker histone subtypes being knocked out, expression of only a limited number of specific genes gets perturbed [31]. Thus, it appears that different linker histone variants regulate distinct set of genes. Recent genome-wide H1 occupancy studies have further elaborated non-random positioning of somatic linker histones in the chromatin. For example, H1x associates with RNA pol II enriched regions [32, 33]. H1.0 is present in nucleolus-associated domains; H1c is enriched at regions having low GC-content and lamina-associated domains (LADs); and linker histone H1a is enriched at intragenic-CpG islands [32–34]. Furthermore, histone H1b binds to blocks of genic and intergenic regions in differentiated cells [35]. However, the genomic occupancy of any of the testis-specific linker histones is currently not known.

Mammalian spermatogenesis witnesses many chromatin remodeling events with unique chromatin reorganization during mammalian spermiogenesis where most of the canonical histones are replaced by histone variants and transition proteins (TPs) in the elongating spermatids, and finally replaced by highly basic proteins, protamines (PRMs), which occupy majority of the chromatin in spermatozoa [36, 37]. Testis-specific linker histone variants appear during different steps of spermatogenesis. For example, linker histone H1t appears in the mid-pachytene spermatocytes and continues to express in round spermatids (step 8 of mouse spermiogenesis) [38, 39]. In vitro DNA condensation studies using CD spectroscopy have shown that H1t is a poor condenser of DNA and chromatin [7, 8]. Linker histone H1T2 appears during step 5 of mouse spermiogenesis and continues until step 15 spermatids. Further, H1T2 is proposed to be involved in the replacement of histones and the formation of nucleo-protamine complex in the mature sperm [14, 15, 17, 40]. Linker histone HILS1 expresses during steps 9–15 of mouse spermiogenesis [6, 41]. Since the expression of HILS1 overlaps with the replacement of histones by TPs and PRMs, it is possible that HILS1 may play an important role in facilitating the histone/protamine transition process. However, there has been no study addressing the function of HILS1 during spermiogenesis. In this study, we have analyzed the secondary structure and chromatin condensation properties of HILS1 in comparison with other linker histones. Further, we studied the genomic occupancy of HILS1 to gain insights into its role in chromatin restructuring process during mammalian spermiogenesis.

## Results

### Linker histone HILS1 possesses less α-helicity compared to H1t and H1d

**Fig. 1:**
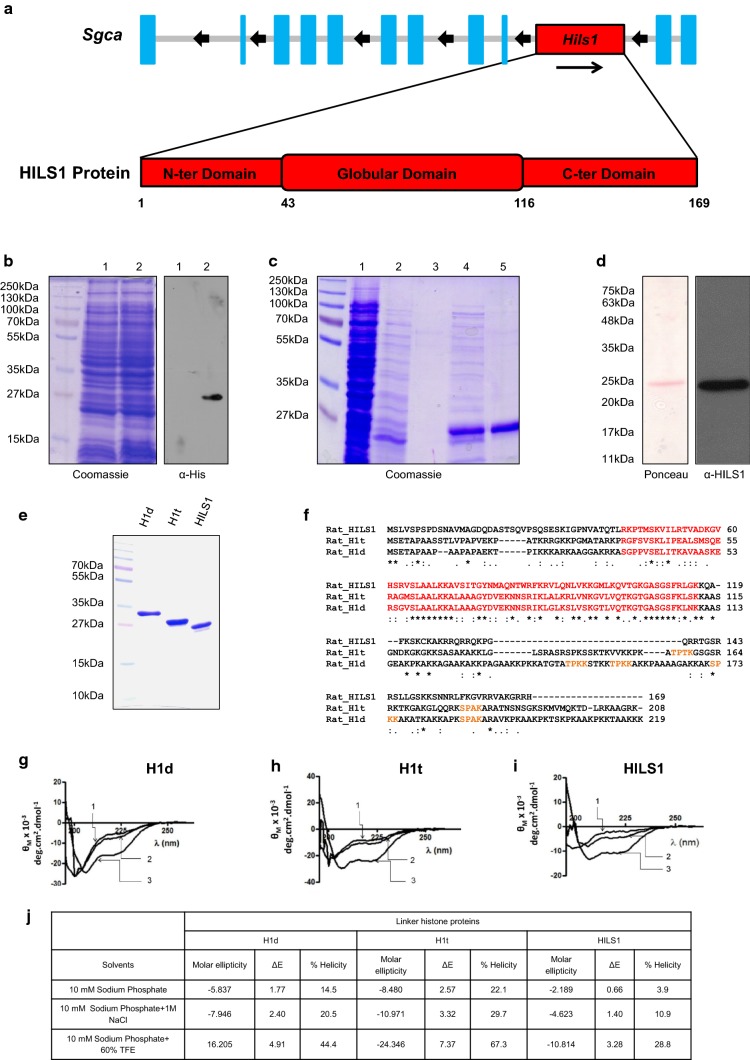
Secondary structure of linker histone H1 variants H1d, H1t, and HILS1. **a** Schematic representation of *Hils1* gene on rat chromosome 10q31 located within the intron 9 of the α-sarcoglycan gene (*Sgca*). HILS1 protein consists of 169 amino acid and has three predicted domains similar to other linker histones that include N-terminal domain (NTD: 1–42 amino acid residues), globular domain (GD: 43-115 amino acid residues), and C-terminal domain (CTD: 116-169 amino acid residues). **b** Over expression of His-tagged HILS1 protein after induction with 0.1 mM IPTG. Lane 1 and lane 2 represents total cell extract from un-induced and 0.1 mM IPTG induced *E. coli* culture for 12 h at 18 °C, respectively. Protein expression was confirmed by western blotting analysis with anti-His (H1029, Sigma, WB: 1:1000) antibodies. **c** Coomassie-stained image of a 12% SDS-polyacrylamide gel depicting fractions collected at different steps of purification of recombinant HILS1 (rHILS1) by Ni-NTA affinity column chromatography. *E. coli* Rosetta(DE3)pLysS cells harboring the plasmid pHILS1 were induced with 0.1 mM IPTG for 12 h and purified as described in methods. Lane 1 represents total cell extract of *E. coli* Rosetta(DE3)pLysS cells expressing HILS1, lane 2 represents proteins not bound to Ni-NTA column, lanes 3, 4 and 5 represent proteins eluted with elution buffer containing 30, 50 and 80 mM imidazole, respectively. HILS1 protein migrates at ~ 25 kDa (lane 5). **d** Western blot analysis of purified HILS1 protein showing a single band at ~ 25 kDa with anti-HILS1 antibodies. **e** Coomassie-stained image of a 12% SDS-polyacrylamide gel showing purified recombinant His-tagged rat H1d, H1t, and HILS1 proteins. Rat H1d and H1t proteins were purified using Ni-NTA agarose column followed by binding with heparin agarose and HILS1 protein was purified by one-step Ni-NTA affinity chromatography as described in methods. **f** Sequence alignment of rat linker histones H1d, H1t, and HILS1, aligned by Clustal Omega software. Globular domain has been highlighted in red. S/TPXK motif in the CTD has been highlighted in orange. Rat H1d possesses four S/TPXK motifs, rat H1t has two, whereas rat HILS1 lacks S/TPXK motif in the CTD. **g**–**i**, Circular dichroism spectra of rat recombinant linker histones H1d, H1t, and HILS1, respectively. A 200 µg/ml solution of purified histone H1 subtypes was used for recording the CD spectrum in a Jasco J-810 spectropolarimeter. The spectra were recorded in 10 mM sodium phosphate buffer, pH 7.5; 10 mM sodium phosphate buffer, pH 7.5 containing 1 M NaCl; and 10 mM sodium phosphate buffer, pH 7.5 containing 60% trifluoroethanol. **j** Summary of α-helicity induced in rat H1d, H1t, and HILS1 in different buffers

Recombinant rat *Hils1* gene is located on chromosome 10, within the 9th intron of the α-sarcoglycan gene and codes for 169 amino acid protein. Sequence comparison with chicken linker histone H5, for which the globular domain structure is known, indicates the presence of NTD comprising of 1st to 42nd amino acids, GD spanning from 43rd to 116th amino acids, and a highly basic CTD consisting of 117th to 169th amino acids (Fig. 1a). Recombinant HILS1 protein was expressed in *Escherichia coli* cells with 6X Histidine-tag at the C-terminus. Expression of rHILS1 protein was confirmed by Western blot analysis with anti-His antibodies (Fig. 1b). HILS1 protein was purified by using Ni-NTA agarose beads (Fig. 1c, lane 5) and was detected as migrating at ~ 25 kDa. Identity of purified HILS1 protein was confirmed by Western blot analysis with anti-HILS1 antibodies (Fig. 1d). To further confirm the identity of the purified protein, we performed MALDI-TOF MS analysis. Database search using MASCOT indicated 79% peptide coverage for rat HILS1 protein (Table 1).

**Table 1:** HILS1 peptides identified by MALDI-TOF MS

Start-end	Observed	Mr (expt)	Mr (calc)	Delta	Peptide
64-89	2969.9070	2968.8997	2968.6069	0.2929	R.VSLAALKKAVSITGYNMAQNTWRFKR.V + Oxidation (M)
98-127	3226.0170	3225.0097	3225.7954	− 0.7857	K.GMLKQVTGKGASGSFRLGKKQAFKSKCKAK.R
101-127	2283.7210	2282.7137	2283.2634	− 0.5496	K.GASGSFRLGKKQAFKSKCKAK.R
118-132	1948.6210	1947.1173	1947.1173	0.4964	K.QAFKSKCKAKRRQRR.Q
130–55	3051.9900	3050.9827	3050.7258	0.2569	R.QRRQKPGQRRTGSRRSLLGSKKSNNR.L
133–161	3311.5850	3310.5777	330.9035	− 0.3257	R.QKPGQRRTGSRRSLLGSKKSNNRLFKGVR.R
140–158	2149.6670	2148.6597	2148.2239	0.4358	R.TGSRRSLLGSKKSNNRLFK.G
151–162	1475.4370	1474.4297	1473.8640	0.5657	K.KSNNRLFKGVRR.V

Since HILS1 is a newly reported linker histone variant whose structure and function are not known, we were curious to understand the secondary structure of HILS1 and compare it with well-characterized linker histones H1d and H1t. Multiple sequence alignment of rat H1d, H1t, and HILS1 proteins revealed significant divergence particularly in the N- and C-terminal domains. Rat HILS1 and H1t proteins showed amino acid sequence similarity of 30.18%, while rat HILS1 and H1d exhibit 30.77% sequence similarity (Fig. 1f). Although GD is the most conserved domain among linker histones, GD of rat HILS1 showed only 48.65 and 43.24% similarity with H1d and H1t, respectively. It has been reported before that globular domain of linker histones possess a defined structure (winged-helix fold) in high-salt condition (> 150 mM NaCl) and further attains secondary structure in the unstructured NTD and CTD in the presence of trifluoroethanol (TFE, an α-helicity inducer/stabilizer) or macromolecules like DNA [42–44]. Since analysis of the amino acid sequence of HILS1 showed high degree of diversity even in the GD, we were curious to know the extent of α-helicity in HILS1. For this, we purified rat rH1d, rH1t, and rHILS1 proteins from *E. coli* (Fig. 1e) and recorded the CD spectra in three different conditions: (1) in the presence of 10 mM sodium phosphate, pH 7.5; (2) 10 mM sodium phosphate, pH 7.5 containing 1 M NaCl and (3) 10 mM sodium phosphate, pH 7.5 containing 60% trifluoroethanol (Fig. 1g–i). A summary of percentage α-helicity induced in all three linker histones under different buffer conditions are shown in Fig. 1j. Percentage α-helicity observed in HILS1 in sodium phosphate buffer was 3.9%, compared to 14.5% in H1d, and 22.1% in H1t. In 1 M NaCl, α-helicity of linker histones increases [43, 45–47] and in our study α-helicity increased to 10.9% for HILS1, 20.5% for H1d and 29.7% for H1t. This increase confirms that rHILS1 is able to attain helicity in 1 M salt as reported for other linker histones previously [48]. As TFE stabilizes the intra-molecular H-bonds due to its relatively poor H-bonding capacity, it works as an α-helicity inducer and stabilizer. Δ*ε*_220,_ which determines the α-helicity, increases with increase in TFE concentration up to 65%, and above this concentration, the protein precipitates [43, 47]. To avoid any possible precipitation, we studied α-helicity in phosphate buffer containing 60% TFE and observed significant increase in α-helicity for all the three proteins (28.8% in HILS1, 44.4% in H1d, and 67.3% in H1t). Comparing these data reveals that HILS1 has the least α-helicity among these proteins in all three conditions tested. This significantly low α-helicity can be attributed to high diversity of amino acids in the primary structure of HILS1 protein and suggests that it is a more disordered protein compared to other histone H1 subtypes, which was also consistent with analysis using PrDOS (Protein disorder prediction system) (data not shown) [49].

### HILS1 is a poor condenser of oligonucleosomal DNA

**Fig. 2:**
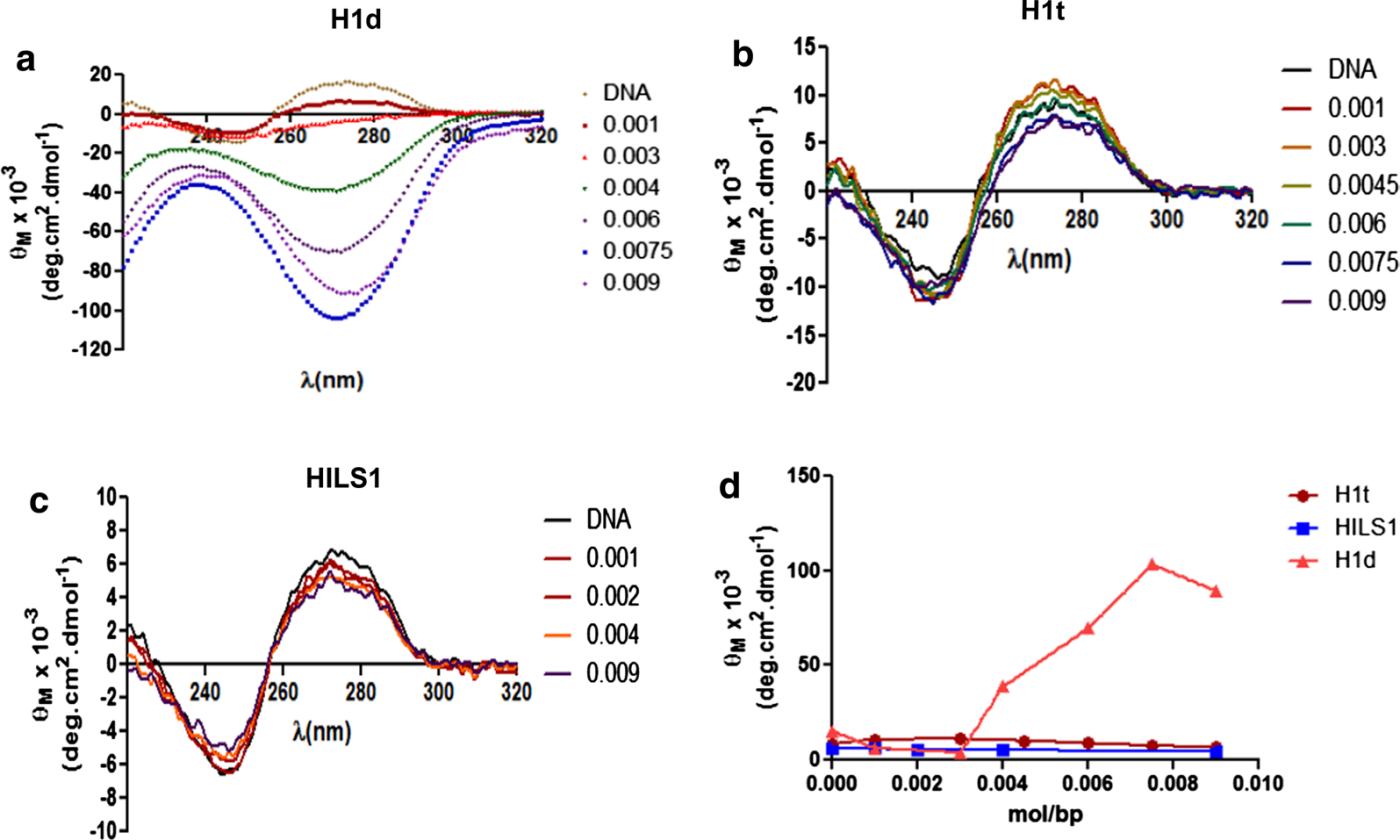
Circular dichroism spectra of histone H1-DNA complexes. Rat testis nuclei were digested with MNase (1 Sigma unit/100A_260_ units of nuclei) for different times at 37 °C. The DNA was purified and analyzed on 1.5% agarose gel in TAE buffer. Oligonucleosomal DNA corresponding to 0.5–2 kb was electroeluted and used for CD experiments. **a**–**c**, Spectra obtained with linker histone H1d, H1t, and HILS1-oligonucleosomal DNA complex, respectively. The spectra of the various protein–DNA complexes prepared at different histone H1/DNA ratios, as mentioned in the text, in 150 mM NaCl, 10 mM Tris–HCl pH 7.4, 0.1 mM EDTA, were recorded in a JASCO 810 spectropolarimeter. Histone/DNA ratio has been shown on the right side of the spectra. **d** Comparison of the in vitro DNA condensation property of rH1d, rH1t, and rHILS1 protein. Ellipticity changes observed in figures** a**–**c** with rat oligonucleosomal DNA upon binding to histone H1, termed, Δ*θ* are plotted as a function of protein/DNA ratio

In order to understand the DNA and chromatin condensation property of the CTD of HILS1, we compared the primary structure of HILS1 with H1t (having poor DNA and chromatin condensation property) and H1d (having strong DNA and chromatin condensation property) (Fig. 1f) [8]. It is well documented that ‘S/TPXK’ motif in the CTD of H1 is responsible for DNA and chromatin condensation. Upon comparing the CTD of rat HILS1, H1d, and H1t, interestingly we observed that HILS1 doesn’t possess any such motif as opposed to four such motifs in the H1d and one motif in the H1t. An earlier study showed the weaker DNA binding ability of HILS1 in comparison with H1T2 than histone H1c [40]. To further understand the DNA condensation property of HILS1 protein in vitro, we performed CD spectroscopy using DNA isolated after MNase digestion of rat testis nuclei. CD spectroscopy has been a preferred method to study DNA and chromatin condensation in vitro [20, 23, 50]. We recorded the circular dichroism spectra of the DNA mixed with increasing amount of different histone H1 subtypes (Fig. 2a–c). It has been reported that at moderate salt concentrations, binding of linker histones to DNA distorts the CD of DNA giving rise to a *Ψ* type of spectrum [51]. In this study also, addition of increasing amount of linker histones progressively decreased the positive *θ*_270 nm_ and generated the* ψ* type of spectrum. Although all the subtypes generated *ψ* type of spectrum, the absolute value of negative *θ*_270 nm_ generated varied with the type of linker histone variants being used. The decrease in *θ*_270nm_ upon addition of each histone H1 to free DNA was plotted against *r*_value_ (protein/DNA ratio, mol/bp), and it revealed that the net condensation observed with saturation level of HILS1 was much lower than observed for histone H1d but was similar to H1t (Fig. 2d). Similar observations were made earlier in case of H1bdec and H1t [8]. Thus, condensation of DNA in vitro by testis-specific linker histones H1t and HILS1 appears to be very low.

### HILS1 is a poor condenser of chromatin

**Fig. 3:**
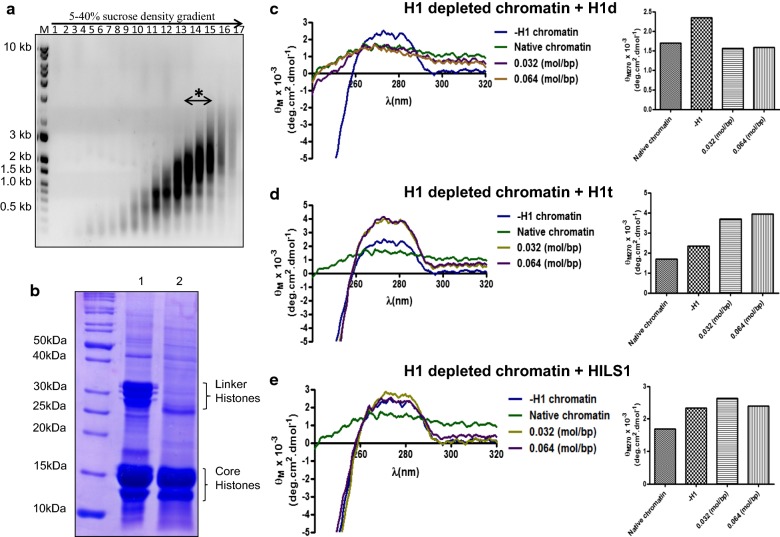
Circular dichroism spectra of H1 depleted chromatin and its complexes with linker histone. Polynucleosomes (10–30 mer) were prepared from soluble chromatin of rat testis by ultracentrifugation on a linear 5–40% sucrose density gradient. Linker histone H1 was stripped off using CM Sephadex beads in buffer containing 350 mM NaCl. **a** Electrophoretic pattern of different fractions of DNA isolated from 5–40% sucrose density gradient ultracentrifugation of MNase digested nuclei in 1.5% agarose gel. Chromatin from 13th–15th fractions (boxed) was used for CD experiments. **b** 12% SDS-PAGE of proteins associated with native (lane 1) and H1-depleted chromatin (lane 2). **c**–**e**, Circular dichroism spectra of histone H1-depleted chromatin after binding with different amount of histone H1 subtype. H1-depleted chromatin was mixed with individual histone subtype in the histone/DNA ratio of 0.0032 (mol/bp); and 0.0064 (mole/bp), respectively, in 80 mM NaCl, 10 mM Tris–HCl, pH 7.4, 0.1 mM EDTA. Spectra were recorded after incubating the chromatin-protein complex overnight at 4 °C. Bar diagram shows the molar ellipticity in different experimental conditions

Since nucleosome is the main site of binding for linker histones in vivo, we were interested to examine the chromatin condensation property of these linker histones in the context of chromatin. For this purpose, oligonucleosomes were prepared by sucrose gradient ultracentrifugation after MNase digestion of rat testicular nuclei (Fig. 3a) and histone H1s were depleted from chromatin (Fig. 3b, lane 2). Linker histone subtypes rH1d, rH1t and rHILS1 were added to the H1-depleted chromatin separately at a ratio of 0.0032 (mole/bp; approximately 1 molecule of H1 per 2 nucleosomes) and 0.0064 (mole/bp; approximately 1 molecule of H1 per nucleosome) [8]. Open chromatin shows high positive ellipticity at *θ*_270 nm_, and ellipticity decreases proportionally with condensation. Positive ellipticity at 270 nm of histone H1-depleted chromatin increased to 2349° cm^2^ dmol^−1^ from that of 1696° cm^2^ dmol^−1^ for native chromatin (condensed chromatin). The CD spectra of the histone H1-depleted chromatin after addition of histone H1d, H1t, and HILS1 are shown in Fig. 3c–e. Addition of histone H1d at a ratio of 0.0032 (mole/bp) and 0.0064 (mol/bp) resulted in a substantial decrease in *θ*_270 nm_ to a value of 1561 and 1583° cm^2^ dmol^−1^, respectively (Fig. 3c). However, addition of similar ratios of histone HILS1 and H1t to H1-depleted chromatin did not result in any significant decrease in the positive ellipticity (Fig. 3d, e). Thus, it appears that unlike H1d, HILS1 protein is unable to condense the chromatin in vitro, a property similar to histone H1t.

### HILS1 associates with intergenic regions of elongating/condensing spermatids genome

**Fig. 4:**
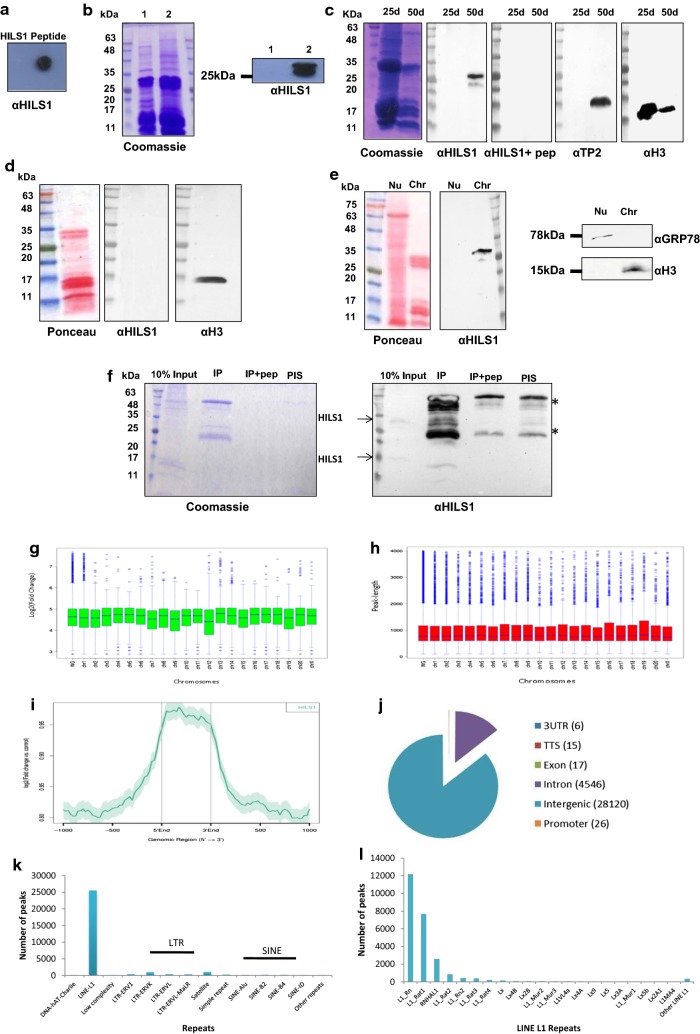
HILS1 primarily associates with intergenic and intronic regions. ChIP sequencing was done using antibody against HILS1 in rat elongating/condensing spermatids. **a** Dot blot analysis of affinity purified antibody against HILS1 with respective peptide. **b** Western blot analysis of the acid extracts from rat liver (lane 1) and testis (lane 2) with antibody against HILS1. **c** Western blot analysis of acid-soluble proteins from 25-day postnatal (lane 1) and 50-day postnatal (lane 2) rat testis nuclei with anti-HILS1 antibodies in the presence or absence of peptide competition with a 200-fold molar excess of the peptide used for antibody generation, as indicated. Western blot analysis with anti-TP2 and anti-H3 antibodies served as a positive control. **d** Western blot analysis of acid extracts of epididymal sperm using HILS1 antibody. **e** Rat testis nuclei were separated into soluble (nucleoplasm; lane 1) and insoluble (chromatin; lane 2) fractions. Western blot analysis was done using antibody against HILS1. Anti-GRP78 antibodies and anti-histone H4 antibodies served as a positive control to probe the soluble fraction (lane 1) and insoluble fraction (lane 2), respectively. **f** Western blot analysis of the pull-down fractions with α HILS1. Input (lane 1), anti-HILS1 antibody (lane 2), anti-HILS1 antibodies + peptide (lane 3), IgG (lane 4). No signal was observed with pre-immune IgG, and heavy and light chains are indicated by asterisk. Antibody specificity is analyzed by confirming the pulled-down bands as HILS1 by mass spectrometry (Additional file 1: Figure S1). **g** Box plot showing the log_2_fold enrichment of HILS1 peaks (*y*-axis) across different rat chromosomes. **h** Box plot representing the HILS1 associated average peak length (*y*-axis) across different chromosomes (*x*-axis) indicating that HILS1 peaks are broad in nature. **i** Distribution of log_2_fold change of the HILS1 aligned reads over the input control that fall within ± 1000 bp of the known 17057 CpG islands in *Rattus norvegicus* genome rn5. **j** Pie chart distribution of HILS1 peaks for various genomic features in rat spermatids. Most of the peaks are bound to intergenic and intronic regions. **k** Bar diagram of number of occurrences of peaks across repeat elements. **l** Bar diagram showing the number of occurrences of peaks across different subclasses of LINE-1 repeat elements

To understand the genome-wide occupancy of rat HILS1 protein in the spermatid chromatin, chromatin immunoprecipitation (ChIP) sequencing was performed in elongating/condensing spermatids using antibody against a unique peptide within the C-terminal domain of HILS1. In order to validate the ChIP-grade quality of the in-house raised antibody [6], additional experiments were performed. Peptide unique to the rat HILS1 CTD with high antigenicity and hydrophilicity was chosen to immunize rabbits, as detailed in Mishra et al. [6]. First, we confirmed the reactivity of the purified antibody to the antigenic peptide by dot blot analysis (Fig. 4a). Further, we confirmed the specificity of the HILS1 antibody by Western blot analysis of acid extracts of testis and liver tissues and we observed the HILS1 protein migrating around 25 kDa only in lane loaded with testis acid extracts (Fig. 4b). This ruled out the possibility of this antibody cross-reacting with any other somatic linker histone subtypes. As reported previously [41], HILS1 protein levels are high in 35–60-day postnatal mice testis when the elongating and condensing spermatids are most abundant, we performed Western blot analysis using our laboratory raised antibody with acid-soluble proteins extracted from 25- and 50-day postnatal rat testis nuclei and as expected we observed band at ~ 25 kDa only in lane loaded with proteins from 50-day postnatal rat testis (Fig. 4c) and no band was observed in the lane loaded with proteins extracted from 25-day postnatal rat testis since HILS1 protein is not expressed at this age, confirming the specificity of this antibody for HILS1 protein. Further, we performed peptide competition experiment by pre-incubating anti-HILS1 antibodies with 200-fold molar excess of the antigenic peptide followed by Western blot analysis, which resulted in complete disappearance of the protein band. This further confirmed that our antibody was highly specific for HILS1 protein. Antibodies against transition protein-2 (TP2) and histone H3 were used as additional controls. TP2 is present in condensing spermatids at the same stage of HILS1 expression. As expected, we observed a single band in western blot analysis with anti-TP2 antibodies, loaded with proteins extracted only from 50-day postnatal rat testis. As H3 is present throughout the spermatogenesis with relatively lesser amounts in elongating and condensing spermatids, Western blot analysis using anti-histone H3 antibody lighted up bands in both lanes loaded with proteins extracted from 25- and 50-day postnatal rat testis, respectively. Consistent with its spermatid-specific expression [41], HILS1 was not detected in the histone extracts of mature sperm collected from rat epididymis (Fig. 4d). In order to confirm the chromatin association of HILS1, rat testis nuclei were fractionated into nucleoplasm and chromatin fractions. Western blot analysis revealed that HILS1 is predominantly associated with chromatin, confirming the earlier observation made in mouse testicular nuclei [41, 52]. Anti-histone H3 antibodies and anti-GRP78 antibodies served as positive controls for chromatin and nucleoplasmic fraction, respectively (Fig. 4e).

Soluble extracts from the elongating/condensing spermatids were subjected to immunoprecipitation by antibody against HILS1, and the ChIP Western blot analysis confirmed that HILS1 is immunoprecipitated with highly specific α-HILS1 antibodies. As reported previously [6], HILS1 is a highly unstable protein, we confirmed the presence of both full length (~ 25 kDa) and degraded protein (15–17 kDa) in both input and IP lanes by mass spectrometry (Additional file 1: Figure S1). Non-specific pre-immune IgG and peptide neutralized HILS1 antibody (incubated with ~ 200-fold molar excess of peptide that corresponds to the epitope recognized by the antibody) were used as negative controls in pull-down experiments. No signal was observed in either IgG or peptide neutralized IP lane (Fig. 4f). Immunoprecipitated DNA was subjected to next-generation sequencing using Illumina HiSeq 1000 system (as described in the methods). Chromosome-wise enrichment analysis showed that the number of peaks was very high in chromosome 2 followed by chromosome 1 and chromosome X, while chromosome 12 had the least number of peaks (Additional file 2: Figure S2). We next investigated the fold enrichment across different chromosomes and observed that the median fold enrichment (log_2_fold enrichment) across different chromosomes ranged from 4.41 (chr 12) to 4.79 (chr 13) while the median fold enrichment across whole genome was 4.64 (Fig. 4g, Additional file 3: Table S2). Further analysis of the HILS1 peak length across different chromosomes revealed that HILS1 binding peaks are broad in nature, an observation that was also made earlier for H1d and H1c proteins [53]. Median peak length across whole genome was 776 bp while the peak length cutoff was given as 100–4000 bp (Fig. 4h; Additional file 4: Table S3). Further, we plotted the distribution of log_2_fold change of the HILS1 aligned reads over the input control that fall within ± 1000 bp of the known 17057 CpG islands in *Rattus norvegicus* genome rn5, considering the center of the CpG as “0”. A total of 48 CpG islands (based on number of CpGs in an island) from Rat genome rn5 assembly were found overlapping with HILS1 peaks, (Fig. 4i, Additional file 5: Table S4), which is comparatively very less in comparison with the total 17057 CpG islands identified in the rn5 assembly. We further investigated whether HILS1 enriched regions were associated with genes, proximal regulatory regions or distal intergenic regions and observed that HILS1 peaks were mostly associated with intergenic regions followed by intronic regions (Fig. 4j). These features were mapped to different chromosomes and observed that peaks associated with intergenic and intronic regions were present on all the chromosomes (Additional file 2: Figure S2). Interestingly, very few peaks were present in exon, 3′UTR, TTS and promoter (Fig. 4j; Additional file 6: Table S5). Of note, Tnp2 and Gas8 TTS were occupied by HILS1. Tnp2 codes for transition protein 2 which is a major transition protein involved in the replacement of histones as well as tight packaging of the spermatid chromatin [37]. Gas8 (growth arrest specific) gene codes for a protein that expresses in adult mice predominantly in the testis and plays an important role in sperm motility [54].

### HILS1 shows unique association with LINE-1 repeats

**Fig. 5:**
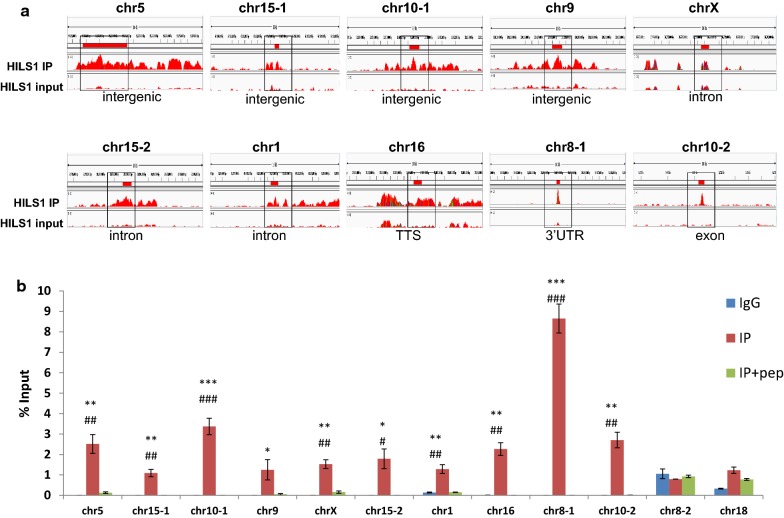
HILS1 shows unique association with LINE-1 repeats. **a** Visualization of selected HILS1 ChIP peaks (10 kb resolution, outlined region represents the exact peaks) generated using IGV genome browser. The *y*-axis unit is read per million (rpm).The *x*-axis represents the genomic positions in base pairs as follows: chr5:95,165,196-95,171,893(MACS_peak_32942-2:chr5); chr15:67,453,898-67,454,808 (MACS_peak_13682:chr15-1); chr10:12,608,450-12,609,577 (MACS_peak_6203:chr10-1); chr9:120,197,981-120,198,900 (MACS_peak_43102:chr9); chrX:36,777,132-36,777,862 (MACS_peak_43949:chrX); chr15:20,798,846-20,802,230 (MACS_peak_12929:chr15-2); chr1:69,879,465-69,880,705 (MACS_peak_1899:chr1); chr16:15,001,197-15,005,927 (MACS_peak_14876:chr16); chr8:39,893,407-39,894,494 (MACS_peak_39650:chr8-1); chr10:3,780,173-3,780,947 (MACS_peak_6054:chr10-2). **b** Quantitative ChIP PCR analysis showing the enrichment of HILS1 across specific regions represented in ChIP-seq peaks (panel A) in the chromatin fraction isolated after pull-down with anti-HILS1 antibodies. Most of the pull-down fractions showed significantly reduced enrichment in the peptide competition control group. Two negative control regions (specific regions of chr 8 and chr 18) were also included for ChIP PCR, where no peaks were present according to the data analysis and visualization. Data were represented as % of input. Results are from three independent experiments, and error bars represent standard deviation. Asterisk shows comparison with IgG control, and # shows comparison with IP+pep group. ***P ≤ 0.001; **P ≤ 0.01; *P ≤ 0.05

Since most of the HILS1 peaks were present in intergenic regions, we then aligned the HILS1 peaks to different repetitive DNA elements present in the UCSC repeat element database and annotated with the help of intersectBed (Bed tools) and HOMER v4.7. We observed that most of the peaks were present in LINE-1 elements, followed by LTR and satellite repeats (Fig. 4k; Additional file 7: Table S6). We also looked for enrichment at 10 kb resolution for selected regions present on different chromosomes and belonging to different categories (Fig. 5a, Additional file 8: Figure S3A and Additional file 9: Figure S3B). Even though we observed some background signals in the input, HILS1 ChIP peaks showed more enrichment signals compared to the input control. To further substantiate the association of HILS1 in LINE-1 elements, ChIP-qPCR assays were employed wherein immunoprecipitation was carried out in the presence or absence of saturating epitope-specific peptide. Primers were designed against the peak regions in different classes of repeat elements. HILS1 immunoprecipitated chromatin showed significant enrichment for all the regions that were HILS1 associated in ChIP-seq analysis such as intergenic, intronic, exon, TTS and 3′UTR regions (especially LINE-1, LTR and satellite repeats) in comparison with the peptide competition group (Fig. 5b). Detailed analysis of HILS1-associated LINE-1 elements revealed the enrichment of HILS1 with different L1 subclasses like L1_Rn, L1_Rat1 and RNHAL1. Among these maximum number of peaks (12,192) are L1_Rn in nature (out of total 56,796 L1_Rn in rn5 assembly; 21.46%), followed by L1_Rat1 (7686) (out of total 19,062 L1_Rat1 in rn5 assembly; 40.32%) and RNHAL1 (2597) (out of total 13,472 L1_Rat1 in rn5 assembly; 19.27%) (Fig. 4l; Additional file 10: Table S7). We further investigated possibilities of DNA sequence-specific HILS1 recruitment to the chromatin in vivo and searched our HILS1 ChIP-seq data for any consensus binding motifs. De novo binding motif search revealed many distinct motifs out of which four distinct motifs that are highly significant are shown in Table 2 (Additional file 11: Table S8). Taken together, these binding motifs are present in 24743 out of 32,731 HILS1-bound peaks, representing 75.59% of the total peaks (Additional file 12: Table S1).

**Table 2:**
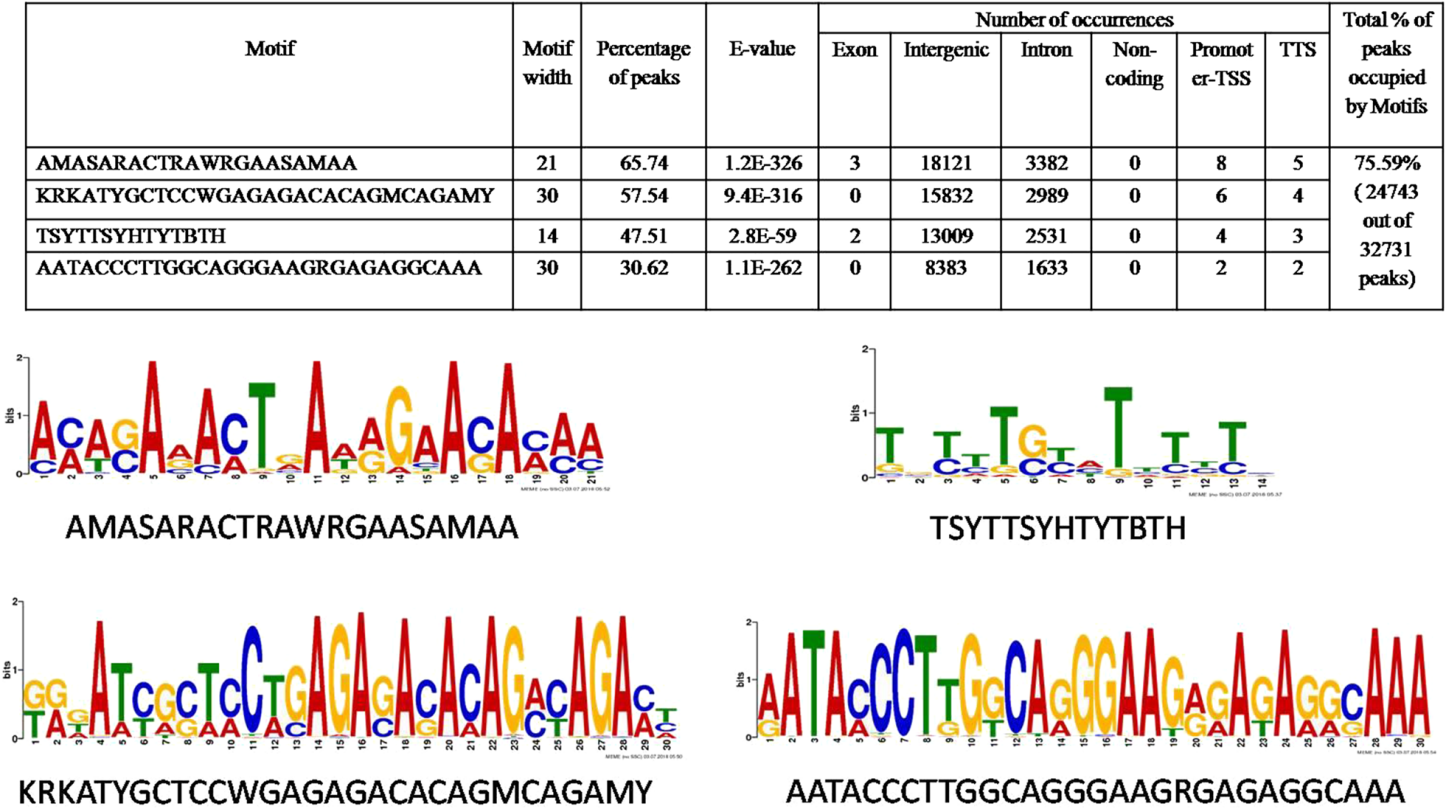
Motif identification and annotation

### Combinatorial histone PTMs identified in HILS1 containing chromatin

**Fig. 6:**
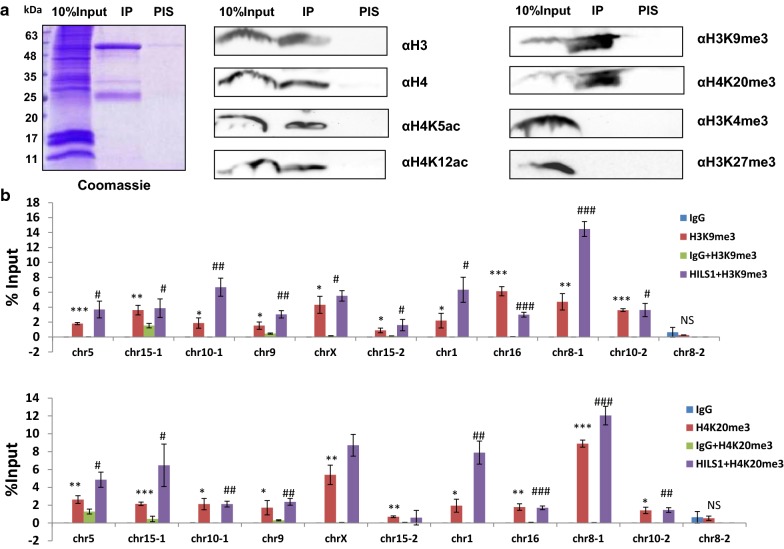
Combinatorial histone PTMs identified in HILS1 containing chromatin in rat elongating spermatids. **a** Western blot analysis of the HILS1 pull-down fractions with antibodies against H4K5ac, H4K12ac, H3K9me3, H4K20me3, H3K4me3 and H3K27me3. Input (lane 1), IP using anti-HILS1 (lane 2), IgG (lane 3). No signal was observed with pre-immune IgG, and the heavy and light IgG chains are indicated by asterisk. Immunoprecipitation showed the specific enrichment of PTMs like H4 acetylation marks, H3K9me3 and H4K20me3 in HILS1 pull-down fraction, whereas methylation marks like H3K4me3 and H3K27me3 were completely absent in HILS1-associated chromatin. **b** Sequential ChIP assays of elongating/condensing spermatids using antibody against HILS1 followed by IP with H3K9me3/H4K20me3 antibody. qPCR analysis showed the specific enrichment of both the modifications in HILS1-associated loci (genomic positions are same as detailed in Fig. 6) in separate IP experiments and the negative control corresponds to a region in chr 8 depleted of HILS1. Data were quantified as % of input. Results are from three independent experiments, and error bars represent standard deviation. Asterisk shows comparison with negative control IgG for single IP, and # shows comparison with IgG+antibody2 for reChIP. ***P ≤ 0.001; **P ≤ 0.01; *P ≤ 0.05

Sperm chromatin possesses unique chromatin organization in comparison with somatic cells, emphasizing the extensive chromatin remodeling during spermatogenesis [55–57]. To gain more insights into the molecular properties of HILS1 in the context of chromatin condensation, we examined whether the HILS1-associated chromatin complex occupies any of the H4 acetylation marks, which provides an open chromatin structure that is necessary for germ cells to progress into spermatogenesis. In line with the CD experiment findings, HILS1 immunoprecipitated fraction showed good enrichment for the histone acetylation marks such as H4K5ac and H4K12ac (Fig. 6a), whose expression is already reported to be high in elongated spermatids to favor histone to protamine substitution [58]. Even though the event of H4 hyperacetylation marks the onset of histone eviction, prior analysis of histones in sperm has largely focused on the overall localization of retained nucleosomes and selected PTMs [59]. Recently, there have been two contradictory reports on the characteristics of the nucleosomes that are retained in the mature sperm. Earlier studies reported that the retained nucleosomes exhibited characteristic histone modifications such as histone H3 lysine 4 trimethylation (H3K4me3) and H3 lysine 27 trimethylation (H3K27me3) and possess high GC content and are significantly enriched at loci of developmental importance, including imprinted gene clusters, microRNA clusters, and HOX gene clusters [60–62]. On the other hand, later studies indicated that the major portion of the nucleosomal binding sites is in repetitive DNA sequences including centromere repeats, short interspersed nuclear element (SINE), and long interspersed nuclear element 1 (LINE-1)—and the majority of nucleosomal binding sites (56.4% in human and 80.2% in bulls) were enriched in distal intergenic regions [63, 64]. While the overall localization of retained nucleosomes is currently a subject of debate, the present finding of locus-specific binding of HILS1 to repeat regions including LINE-1 elements and very low percentage of CpG islands in spermatids is very exciting. However, there have been no reports on specific PTMs in relation to LINE and SINE elements containing nucleosomes in the spermatid genome. In this context, we were curious to examine the associated histone PTMS in the HILS1-bound loci. We performed western blot analysis of the HILS1 immunoprecipitated fraction with antibodies specific for either active or repressive histone modifications. Surprisingly, the IP fraction was more enriched in H3K9me3 and H4K20me3, two well-defined markers of heterochromatin, which were recently reported to be transmitted by the sperm to the embryo [65]. Interestingly, such posttranslationally modified HILS1-bound chromatin is devoid of histone PTMS which are linked to the maintenance of transcription pattern in sperm like histone H3 lysine 4 trimethylation (H3K4me3) and histone H3 lysine 27 trimethylation (H3K27me3) (Fig. 6a, right panel). Single IP experiments demonstrate that DNA repeat sequences (mostly LINE-1) specifically bound to HILS1 are enriched for H3K9me3 and H4K20me3 in spermatids. Sequential ChIP experiments were carried out to further validate the co-occupancy of either of these repressive marks with HILS1 in the repeat regions. For the sequential ChIP, we first precipitated the HILS1-bound chromatin from the elongating/condensing spermatids using antibody against HILS1 and then incubated the eluted fraction with anti-H3K9me3 or anti-H4K20me3 antibodies. ChIPed DNA was quantified by qPCR using specific primers for the regions bound to HILS1 including LINE-1, LTR and satellite repeat regions (Fig. 6b). Results confirmed the co-occupancy of HILS1 with H3K9me3 or H4K20me3 in the selected peak regions of the HILS1 pulled-down chromatin; however, the co-occurrence of both H3K9me3 and H4K20me3 within the HILS1 occupied chromatin domain has not addressed in the present study. Altogether these data show that HILS1-associated chromatin exhibits very unusual characteristics combining H4 acetylation and repressive histone marks, especially at HILS1-bound LINE-1 repeats.

## Discussion

During evolution, mammals and other organisms have acquired the capacity to express specific nucleosomal and linker histones in their male germ cells. However, the role of such testis-specific chromatin components in the processes that occur during spermatogenesis remains unknown. Spermiogenesis is a highly complex differentiation process during which most of the histones are replaced by protamines to compact the genome to highly condensed mature sperm. The present study was undertaken to analyze the role of testis-specific linker histone, HILS1 in the formation of intermediate chromatin structures during histone/protamine transition in spermiogenesis, which might require decondensation of the spermatid chromatin. Among testis-specific linker histones, HILS1 expression begins in early elongating spermatids and continues to express in condensing spermatids [6]. The first report on the presence of linker histone HILS1 in spermatids of mouse and human came a decade ago showing that HILS1 is a chromatin-bound protein and represents around 10% of all chromatin proteins present in the spermatids [41, 52]. HILS1 is present in the testis when the nucleosomal structure of the chromatin is lost and most of the DNA is bound to highly basic proteins such as transition proteins and protamines [41, 52]. HILS1 was shown to bind to reconstituted mononucleosomes and also produces chromatosome stop during MNase digestion, although its binding to the mononucleosome appeared less efficient than somatic linker histone H1a [41]. Previous study from our laboratory found that rat HILS1 exhibits extensive posttranslational modifications that include many phosphorylation and acetylation sites in all the three structural domains [6]. In the present study, western blot analysis using antibody against CTD of HILS1 confirmed that the HILS1 expression is confined to elongating and condensing spermatids and later disappears in the mature sperm (Fig. 4), which rules out the possibility of direct transmission of the HILS1-associated chromatin complex to embryo. Because HILS1 has diverged significantly from other linker histone family members, it may have multiple functions in the chromatin remodeling during spermatogenesis. The current work identifies the two possible independent levels of biological roles of HILS1, a) favors histone eviction required for TP/protamine loading by loosening or maintaining the nucleosome-bound chromatin b) HILS1 is involved in the epigenetic preprogramming of the spermatid genome for embryo development.

Linker histones possess ability to bind to DNA and form soluble aggregates as a result of DNA condensation. Our laboratory had reported earlier that condensation property of linker histones is localized in octapeptide repeats containing S/TPXK motif in the CTD of the H1 proteins [21]. This was further supported by another study using mutational approach reporting that the ability of histone H1^0^ to stabilize chromatin folding was localized in two specific subdomains of 24 amino acids in the CTD, containing S/TPXK motifs [19]. These sequences mediate CTD-dependent condensation of naked DNA by forming β-turns that bind the minor groove of DNA [23, 24, 66–68]. Previous modeling studies from our laboratory have demonstrated that the CTD has the propensity to adopt an HMG-box-like structure and that the CTD S/TPXK motifs are the sites of DNA binding and function in the compaction of the DNA [20, 23, 67]. Importance of the CTD in binding with chromatin has also been studied by fluorescence recovery after photobleaching (FRAP) experiments where deletion of specific region of CTD containing S/TPXK motif resulted in decreased binding affinity compared to wild-type protein [69]. Further using FRAP, a correlation between H1 CTD length and binding affinity was drawn, whereby linker histones having longer CTD bind tightly to the chromatin and exchange slowly (over a period of ~ 15 min) [70]. However, linker histones having short CTD have a recovery time of only ~ 1–2 min [71]. Notably, multiple sequence alignment of the amino acids corresponding to rat H1d, H1t, and HILS1 revealed that HILS1 has the shortest CTD (53 amino acid residues) among all eleven linker histone subtypes and it also lacks any S/TPXK motif (Fig. 1f). Thus, we speculated that HILS1 may behave differently in the nucleus and should bind loosely to the chromatin. Earlier studies have reported that rat H1t, having only one S/TPXK motif but lacking octapeptide S/TPXK repeat in the CTD, has poor ability to condense DNA and chromatin, while rat H1d having four S/TPXK motifs condenses DNA and chromatin strongly [8, 21, 23]. Notably, we have observed in the present study that HILS1 having no S/TPXK motif in the CTD also showed poor condensation of the DNA and chromatin (Figs. 2, 3). Also, in HILS1 many amino acids are substituted with other residues in the previously reported two 24 amino acids stretches in the CTD, which are essential for the chromatin and DNA condensation [19]. Thus, H1t and HILS1 may behave similarly in maintaining open chromatin structure at different stages of spermatogenesis. It is very interesting to point out here that HILS1 appearance in spermatid chromatin occurs at the same time when histone H1t disappears. It is possible that HILS1 may provide a favorable open chromatin structure to the spermatid at specific loci for the recruitment and deposition of other basic proteins during histone replacement in mammalian spermiogenesis. However, we would like to highlight that we cannot ascertain at present whether such an open chromatin structure is manifested by HILS1 in vivo in the spermatid chromatin.

Numerous studies suggest that the genome undergoes regional differentiation during mammalian spermiogenesis and the exact molecular mechanism underlying this process is unknown. This sequence-specific chromatin conformation has been suggested to facilitate transcriptional activation of paternal genes in early embryogenesis and enable the three-dimensional organization of the sperm nucleus [72–74]. Genome-wide occupancy analysis of HILS1 in rat elongating/condensing spermatids showed that HILS1 is bound preferentially to the intergenic and intronic regions of the genome (Fig. 4). Our data show the preferential association of HILS1 with LINE-1 elements (majorly L1_Rn) followed by LTR and satellite repeat elements. In contrast, a significant depletion of HILS1 occupancy is observed at important regulatory regions particularly exon, 3′UTR, promoter-TSS and TTS. Notably, HILS1 enrichment is not restricted to the intergenic/intronic regions, but it also showed very low enrichment with CpG islands (Fig. 4i). This opens up the possibility that HILS1 occupancy with CpG islands represents a small subset of HILS1-bound regions in the rat elongating spermatid genome. Overall findings highlight the specific binding of HILS1 to the intergenic and intronic regions of the elongating spermatid genome similar to most of the somatic H1s, as supported by the motifs identified for HILS1 using de novo motif binding search [34]. The knockout study has indeed revealed that linker histones regulate DNA methylation and thus promotes epigenetic silencing [30]. Notably, in sperms, few genomic regions in the nuclear periphery retain nucleosomal structure and mainly localize to un-methylated CpG islands in a histone variant–specific manner, although the role of HILS1 in nucleosomal retention or methylation status of the bound loci was not addressed [61].

Direct consequences of incorporation of testis-specific histone variants in spermatogenesis have been summarized recently to include open chromatin, nucleosome instability, histone disassembly to facilitate the histone–protamine transition [75]. The most dynamic change occurs through the hyperacetylation of histone tails from meiosis through the round and elongating/condensing spermatid stages, followed by the subsequent loss of acetylated histones in mature sperm [76, 77]. An interesting question arising in this area is that whether histone acetylation events create an open chromatin structure and mediate the insertion of testis-specific histone variants or vice versa. As mentioned in Results section, HILS1 (being the poor condenser of chromatin)-associated chromatin is found to be enriched in histone acetylation marks like H4K5ac and H4K12ac, which has previously been detected in round and elongating spermatids [78, 79]. Nucleosomal retention is another one important event occurring in elongating spermatids, and there is contradictory observations made by different groups in the genomic loci associated with these retained nucleosomes in sperm [62, 63]. Current understanding of nucleosomal retention in sperm is dominated by the finding that nucleosomes retained in CpG-rich promoters represent a small fraction of all nucleosome retained in mature sperm which occurs robustly across repeat sequences of gene poor regions rather than developmental promoters [63, 64]. However, based on previous studies on H4K20me3, it has been suggested that a small percentage of the genomic DNA remains packaged with histones in mature mouse sperm [80] can be protected against paternal DNA demethylation in the zygote [81]. An independent study reported that paternal heterochromatin formation in human embryos is H3K9/HP1 directed and primed by sperm-derived histone modifications [65]. Interestingly Delaval et al. [81] have reported the unique combination of high levels of H4K20 methylation and H4 acetylation in LINE-1 elements during late spermatogenesis. Remarkably, the detailed analysis of HILS1 ChIP-seq data has shown the preferential enrichment of HILS1 at LINE-1 sequences unlike other linker histones (Figs. 4, 5) [53]. Immunoprecipitation studies demonstrate that HILS1-bound chromatin loci are also enriched in heterochromatin specific modifications like H3K9me3 and H4K20me3, whereas transcription pattern-specific modifications like H3K4me3 and H3K27me3 are not associated with HILS1 in sperm. Sequential ChIP and qPCR experiments further confirmed the findings that co-occupancy of HILS1 with either of the heterochromatin specific histone modification marks like H3K9me3 or H4K20me3 represents a consistent feature of the HILS1-bound loci (predominantly LINE-1 repeats) (Fig. 6), which may represent a subset of spermatid chromatin bound to heterochromatin specific modifications. Apart from this, unusual combination of H4 acetylation and heterochromatin specific modifications like H3K9me3/H4K20me3 in the HILS1-bound chromatin represents a distinct behavior of spermatid chromatin, which is already reported as specific feature of pericentric heterochromatin during post-meiotic chromatin reorganization in male germ cells [79, 82]. Recent studies are paying more attention to LINE-1 elements, as they become reactivated from parental genomes shortly after fertilization in zygotes and are reported to be a potential player in gene regulation in early embryogenesis [83, 84]. Taken together, we suggest that HILS1 incorporation coincides with the event of histone hyper acetylation results in the locus-specific formation of specific higher-order chromatin structures encompassing LINE-1 repeats, which may play an important role in shaping the chromatin landscape of embryo within the broad context of global chromatin remodeling of the paternal genome during spermiogenesis. Further studies on the nucleosomal retention and methylation status of HILS1-bound loci will greatly improve our understanding of the involvement of HILS1 in the process of preprogramming of the paternal genome.

## Conclusions

In this study, we have analyzed the secondary structure of HILS1 by circular dichroism spectroscopy and demonstrated that it has less α-helicity compared to somatic histone H1d and testis-specific histone H1t. Further, we show that HILS1 is a poor condenser of DNA and chromatin compared to somatic H1d. Genome-wide occupancy analysis revealed that HILS1 predominantly associates with the intergenic and intronic regions, preferentially LINE-1 elements of the rat spermatid genome. This behavior points out toward the potential role of HILS1 in the maintenance of epigenetic signatures favouring preprogramming of the paternal genome to determine its differential contribution to the early development and trans-generational inheritance.

## Methods

### Genomic organization of *Hils1* and multiple sequence alignment

Information on the genomic organization of the rat *Hils1* gene (NCBI gene ID: 690026, mRNA: NM_001109565.1) was obtained from the NCBI database. Domain organization of this protein was constructed based on the sequence comparison with chicken H5 (Uniprot Id:P02259) [6]. Multiple sequence alignment was performed by Clustal Omega software using rat H1d (Uniprot Id: P15865), rat H1t (Uniprot Id: P06349), and rat HILS1(Uniprot Id: D3ZZW6) amino acid sequences.

### Cloning of rat *Hils1* in pTrc99A(+) prokaryotic expression vector

Total RNA was extracted from 50-day postnatal rat testis using TRIzol reagent (Invitrogen) and reverse transcribed by the SuperScript^TM^III reverse transcriptase (Invitrogen) using oligodT primers. Rat *Hils1* gene was amplified from rat testis cDNA using gene-specific primers with NcoI and BamHI restriction endonuclease sites as linkers in the sense and antisense primers, respectively, for the directional cloning. The primers used for PCR amplification were: *Hils1* sense primer (5′-CATGCCATGGCGCTGGTGTCACCATCTCCAG-3′); *Hils1* antisense primer (5′-CGGGATCCTTAGTGATGGTGATGGTGATGGCGGCGGCCTTTAGCCACCCTAC-3′) that included 6×His tag to facilitate the purification of the recombinant protein by Ni-NTA affinity column chromatography. The Ser2 (TCT) at the N-terminus was changed to alanine (GCT) to incorporate the NcoI site for cloning. *Hils1* gene was cloned into pTrc99A(+) vector between NcoI and BamHI sites. pTrc99A(+) plasmids with rat H1d and H1t gene were obtained as described previously [15, 48].

### Expression and purification of recombinant HILS1 protein

The Rosetta(DE3)pLysS strain of *E. coli* carrying the expression plasmids for rat *Hils1* was grown in LB medium containing ampicillin (100 μg/ml) and chloramphenicol (34 µg/ml) at 37 °C. Bacterial cultures were grown until optical density (A_600_) reached 0.6 and the expression of HILS1 was induced by 0.1 mM IPTG. The culture was further incubated for an additional period of 12 h at 18 °C. Cells were collected by centrifugation at 6000×*g* for 10 min. HILS1 protein expression was confirmed by Western blot analysis with anti-His (H1029, Sigma, WB: 1:1000) antibody. Recombinant HILS1 was purified by a modified one-step Ni-NTA column chromatography established in our laboratory. Briefly, the cell pellet was resuspended in 6 volumes of lysis/binding buffer (100 mM Tris–HCl pH 7.4, 2 M NaCl, 20% ethanol, 2 mM DTT, 10 mM sodium metabisulphite, 1% Triton X-100, 1 mM PMSF, 1 mM EDTA and 1X protease inhibitor cocktail (Roche)). Lysozyme was added to a final concentration of 1 mg/ml and incubated on ice for 30 min with occasional mixing. The cell suspension was sonicated for 30 min at 40% amplitude, 15 s on and 15 s off cycles on ice. The sonicated lysate was centrifuged at 16,000×*g* for 30 min at 4 °C. The supernatant was mixed with Ni-NTA agarose beads (Invitrogen) pre-equilibrated with lysis buffer. The mixture was kept for binding on an end-over-end rotator for 3 h at 4 °C. After binding, the mixture was transferred to a fresh column and the unbound material was allowed to pass through. Proteins bound to Ni-NTA beads were washed sequentially with 20 column volumes of the binding buffer, binding buffer containing 30 mM imidazole, and finally with the binding buffer containing 50 mM imidazole. Bound proteins were eluted with the elution buffer (50 mM Tris–HCl, pH 7.4, 150 mM NaCl, 1 mM PMSF, 80 mM imidazole, and 1X protease inhibitor cocktail). Proteins from each step of purification were loaded onto a 12% SDS-PAGE, coomassie stained, and analyzed for purity. Purified HILS1 protein was dialyzed against 0.5% acetic acid and lyophilized in aliquots. A total of 1.2 mg HILS1 protein was purified per liter of induced culture. Rat H1d and H1t proteins were expressed and purified as described previously [15, 48]. Quantitation of the HILS1, H1t, and H1d proteins was performed by taking absorbance at 230 nm, A_230_ = 4.2, corresponds to 1 mg/ml for histone proteins [85].

### Mass spectrometry

Mass spectrometry was performed as described previously with minor modifications [37, 86]. HILS1 protein band was excised, and gel pieces were washed with 100 μl of 100 mM ammonium bicarbonate buffer, pH 8.5 for 15 min, dehydrated in 50 μl of acetonitrile at room temperature for 15 min and dried in SpeedVac for 10 min. The gel particles were rehydrated with 50 μl of 10 mM DTT in 100 mM ammonium bicarbonate buffer and incubated at 56 °C for 1 h. The gel pieces were dehydrated twice in 50 μl of acetonitrile at room temperature for 10 min and dried in a SpeedVac for 10 min. Subsequently, 50 μl of 55 mM iodoacetamide in 100 mM ammonium bicarbonate buffer was added and incubated in the dark at room temperature for 45 min to alkylate cysteine residues. The gel pieces were washed with the ammonium bicarbonate buffer and incubated with 100 μl of the same solution at room temperature for 15 min. Further, gel pieces were dehydrated with 50 μl of acetonitrile for 15 min and dried completely in the SpeedVac. Sequencing grade trypsin (150 ng) was added to gel pieces and after digestion at 37 °C overnight, the resulting proteolytic peptides were subjected to hydrophobic extraction (50 μl of 50% ACN, 5% formic acid), and the extracted peptides were analyzed by mass spectrometry. To identify the protein, spectra were analyzed by MASCOT software using NCBInr database. The search parameters included up to four missed cleavage sites, variable oxidation states of methionine, and carbamidomethyl as fixed modification.

### Preparation of rat testis oligonucleosomal DNA and depletion of linker histones from chromatin

Oligonucleosomal DNA and histone H1 stripped chromatin from testicular nuclei were prepared according to the method described previously [8]. Briefly, oligonucleosomes from rat testis were obtained by MNase digestion and fractionated on a linear 5% to 40% sucrose density gradient in a Beckman ultracentrifuge (SW41Ti rotor, 1,97,867×*g* for 22 h). DNA was isolated from different fractions and analyzed by electrophoresis on a 0.8% agarose gel. The fractions containing DNA ranging from 0.5 to 2 kb were purified by electroelution of DNA from the agarose gel. Histone H1 and non-histone proteins were removed from the chromatin by incubating the chromatin in 10 mM Tris–HCl, pH 8.0, 0.1 mM EDTA, 350 mM NaCl with CM Sephadex beads overnight. Supernatant containing H1-stripped chromatin was analyzed on a 12% SDS-PAGE.

### Circular dichroism studies

Circular dichroism studies of protein secondary structure were performed according to the procedure described earlier [48]. Briefly, protein spectra were recorded in following buffers (a) 10 mM sodium phosphate buffer, pH 7.5, (b) 10 mM sodium phosphate buffer, pH 7.5 containing 1 M NaCl and (c) 10 mM sodium phosphate buffer, pH 7.5 containing 60% trifluoroethanol in a Jasco J-810 spectropolarimeter. The α-helical content in different histone H1 subtypes was estimated using the formula [*θ*] = 3298 Δ*ε*, and % helix = (Δ*ε*_220_ − 0.25)/0.105 [47, 48]. DNA condensation and chromatin condensation studies using circular dichroism were performed as described elsewhere [8, 21].

### Extraction and purification of histones from rat tissues

Linker histone HILS1 and other histones were extracted from rat liver/testis nuclei by the method described previously [87, 88]. Briefly, the purified nuclei were resuspended in 0.4 N H_2_SO_4_, homogenized and incubated on ice for 30 min. The homogenized material was centrifuged for 10 min at 10,000×*g* at 4 °C to pellet the residual chromatin. The extracted proteins present in the supernatant were mixed with 30% TCA (final v/v) and incubated at 4 °C for 30 min to precipitate. Precipitated proteins were recovered by centrifugation at 12,000×*g* at 4 °C for 10 min. Supernatant was discarded and the protein pellet was washed once with ice-cold acetone containing 0.05% HCl (acidified acetone) and three times with ice-cold acetone. The pellet recovered was air dried, dissolved in water, and used for western blot analysis.

### Separation of nucleoplasm and chromatin fractions from rat testis nuclei

Rat (50–60-day postnatal) testis nuclei were separated into nucleoplasmic and chromatin fractions as described previously [37]. Briefly, adult rat testicular nuclei were lysed in 5 volumes of nucleoplasm extraction buffer containing 3 mM EDTA and 0.2 mM EGTA and incubated for 30 min at 4 °C on end-over-end mixer. Nucleoplasmic fraction (supernatant) was obtained by centrifugation at 6500×*g* for 10 min at 4 °C and chromatin obtained as pellet. The chromatin pellet was lysed in high-salt buffer (50 mM Tris–HCl pH 8.0, 2.5 M NaCl, 0.05% NP-40 and protease inhibitor cocktail) for 30 min at 4 °C end-over-end mixer. Lysed chromatin was centrifuged at 16,000×*g* for 10 min at 4 °C. Supernatant containing chromatin proteins and earlier obtained nucleoplasmic fractions were precipitated by 30% trichloroacetic acid (TCA) for 30 min at 4 °C, and the pellet containing proteins were recovered by centrifugation at 12,000×*g* for 10 min at 4 °C. The protein pellet was washed once with ice-cold acidified acetone and three times with ice-cold acetone. Recovered pellet was dried, dissolved in water and stored in aliquots at −20 °C.

### Characterization of HILS1 antibody

Western blot analysis and peptide competition assay were performed as described previously [6]. Briefly, histone proteins were loaded on a 15% SDS-PAGE and transferred onto a nitrocellulose membrane. After transfer, the membrane was blocked using 5% skimmed milk powder in PBST (1XPBS with 0.05% Tween 20) for 1 h at room temperature. Membrane was then incubated with primary antibody overnight at 4 °C on a rocking platform. The unbound antibody was removed by washing the membrane three times with wash buffer (PBST) and probed with an appropriate HRP-conjugated secondary antibody in PBST with 1% skimmed milk powder and incubated for 1 h. The membrane was then washed extensively and developed using the chemiluminescence reagent (ECL) from Thermo Scientific.

Peptide competition assay was performed to check the specificity of HILS1 antibody. Briefly, antibody against HILS1 (raised against CTD peptide of HILS1-KSKCKAKRRQRRQKPGQRRT) was pre-incubated with 200-fold molar excess of the HILS1 peptide used for raising antibody for 3 h at 4 °C. After transferring the histone proteins from SDS-PAGE gel onto the nitrocellulose membrane, the membrane was blocked with 5% skimmed milk powder. Peptide-blocked HILS1 antibodies were allowed to bind with the blotted protein for 1 h at room temperature. The rest of the steps were same as for the Western blot protocol described above.

### Chromatin immunoprecipitation (ChIP) and sequential ChIP assays

Nuclei were prepared from 50–60-day postnatal rat testis and decondensed using 10 mM DTT as described previously [37, 89]. Chromatin immunoprecipitation using anti-HILS1 antibodies was performed as described elsewhere [90]. Briefly, nuclei were fixed using 1% formaldehyde for 10 min at room temperature, quenched with 125 mM glycine for 5 min, harvested and sonicated to generate fragments of 100 to 300 bp. For ChIP, 50 μg of sonicated chromatin was immunoprecipitated overnight with 5 μg anti-HILS1 antibodies. Immunocomplexes were captured on 50 μl protein A Dynabeads. Beads with bound immunocomplexes were washed, eluted and reverse cross-linked at 65 °C overnight, and DNA was recovered using Qiagen PCR purification kit. Input was prepared with 5% of the sonicated chromatin. Sequential chromatin immunoprecipitation or ChIP-reChIP using modification specific antibodies were performed according to Furlan-Magaril et al. [91]. First round of IP was performed as described before using an antibody against HILS1. For the Re-ChIP, complexes from the first ChIP were eluted using 50 µl of 10 mM DTT at 37 °C for 30 min. After elution, the beads were separated and the eluate was diluted 20 times with IP dilution buffer [1% Triton X-100, 2mMEDTA, 150 mMNaCl, 20 mMTris–HCl, (pH8.1); supplemented with protease inhibitor cocktail] and subjected again to the ChIP procedure.

### ChIP-seq data analysis

ChIP and input DNA libraries were prepared using the ChIP-seq Sample Preparation Kit (Illumina). Briefly, 50 ng of ChIP and input DNA were repaired to overhang a 3′-dA and adapters were ligated to the end of DNA fragments. DNA fragments of 100–300 bp were size selected after PCR amplification. Library DNA was purified using Qiagen DNA purification kit. Sequencing was performed with Illumina HiSeq 1000 system using paired-end reads of 101nt length. Sequenced reads containing adaptor sequences were trimmed and filtered with Phred quality threshold as 20. Read pairs falling below 20 bp after clipping were removed. Clean reads were uniquely aligned against UCSC *Rattus norvegicus* genome (rn5, Mar. 2012) using Bowtie2 (version 2.2.3) with no mixed and no discordant parameters [92, 93]. All aligned files were first sorted and then PCR duplicates were removed from mapped reads of the aligned (BAM) files. Mapped reads after sorting and indexing were used for the subsequent peak calling by MACS1.4.2 with input as control and a p value cutoff of 1e-05 [94]. The optimal parameter for broad peak calling in MACS was implemented in the present analysis. Read enrichments/peaks were first called for each replicate with the uniquely mapped reads by MACS1.4.2 with default parameters except the “—nomodel” set to TRUE and effective genome size = 1.96e+09. The correlation co-efficiency (*r*) between replicates was calculated based on the ratio of overlapping region and corresponding peak length of each replicate and was calculated to be 0.813 considering 100 bp overlap between the 2 replicates, for a p value of 1e-05. For finding the overlapping peaks, Bed tools Intersect was used with 100 bp between summits and only overlapping peaks (32,731) were used for subsequent analysis. Chromosome-wise distribution plots were generated using custom R script. Genome browser view for chromosome-wise peak distribution was generated using UCSC genome browser. HILS1 peak distribution was identified at genomic locations enriched in CpG islands, repetitive elements, exons, introns, intergenic regions, 3′UTR and 5′UTR using HOMER v4.7 (for annotation) and Bed tools (for identifying the intersections). Box plots were created using R 3.3.2 statistical software. De novo motif identification was done using 300 bp regions surrounding overlapping peak summits as target set and shuffled set of same overlap sequences as background set with MEME software [95].

### ChIP-qPCR assay

For ChIP, 25 μg of sonicated chromatin was immunoprecipitated overnight with 5 μg anti-HILS1 antibodies or peptide-blocked HILS1 antibodies or pre-immune IgG. Immunocomplexes were captured on 50 μl protein A Dynabeads. Beads with bound immunocomplexes were washed, eluted, and reverse cross-linked at 65 °C overnight, and DNA was recovered using Qiagen PCR purification kit. Input was prepared from 5% of the sonicated chromatin. 2µL input or ChIP sample from the recovered DNA suspension of 50µL was used for performing the qPCR analysis.

Specific primers were designed against the HILS1 peaks obtained from ChIP-sequencing experiment in rat testis nuclei (primers are listed in Additional file 12: Table S9). The enrichment of specific regions of the genome were quantified by qPCR reaction using ChIP DNA (20 ng), 300 nM forward/reverse primers and 2X SensiFAST SYBR^®^ No-ROX mix (5 µl) using Rotor-Gene 6000 Corbett machine. The PCR conditions were as follows: 95 °C for 4 min; 95 °C for 30 s, appropriate annealing temperature for 30 s and 72 °C for 30 s, for 35 cycles in a total volume of 10 µl. The *C*_t_ value (number of cycles required for the fluorescent signal to cross the threshold) was recorded in the experimental report after analysis by Rotor-Gene 6000 software. The *C*_t_ values of the duplicates showed minimal variability, and *C*_t_ values were used for calculating the percent of input using the following formula.

$${\text{Percent}}\,\,{\text{Input}} = 100 \times 2^{{({\text{Adjusted}}\,\,{\text{input}} - C_{\text{t}} \,\,(IP)}} .$$

In sequential ChIP experiments, the amount of genomic DNA co-precipitated with specific antibody was calculated in comparison with the corresponding input DNA used for each immunoprecipitation using the same formula. Primers used for sequential ChIP-qPCR are same as listed in Additional file 13: Table S9.

## Additional files

**Additional file 1: Figure S1.** Mass spectrometric analysis of HILS1 IP bands confirms the specificity of HILS1 antibody. Specificity of the antibody raised against CTD of HILS1 was confirmed by the mass spec analysis of the immunoprecipitated bands. Results represent the peptides identified from 25 kDa and ~15kDa bands detected in western blot in both input and immunoprecipitated lane as represented in Figure 5A. Note that the prominent 25kDa band is the full-length form of HILS1, whereas ~15kDa showed different migration in HILS1 IP lane in comparison with input, which is the cleaved product. Coverage represents the percentage of sequence matching with peptides found in the analysis.

**Additional file 2: Figure S2.** HILS1 mainly associates with intergenic regions across the chromosomes. ChIP sequencing was performed using anti-HILS1 antibodies and analysis of enriched genomic regions was carried out using MACS software. Figure represents the number of peaks associated with different genic elements (y-axis) distributed across different rat chromosomes (x-axis). Most of the peaks are associated with intergenic regions followed by introns across the chromosomes, whereas very less peaks are found in 3′UTR, exon or TSS regions.

**Additional file 3: Table S2.** Chromosome-wise fold enrichment of HILS1 peaks. Excel file represents the fold enrichment values for HILS1 peaks across all chromosomes of the rat genome.

**Additional file 4: Table S3.** Chromosome-wise peak length of HILS1 peaks. Excel file represents the length of HILS1 peaks across the chromosomes confirming their broad nature

**Additional file 5: Table S4.** ChIP peaks (32731) overlapping with UCSC CpG islands. List of CpG islands were obtained from UCSC table browser and used to find overlaps with 32731 HILS1 ChIP peaks using Bed tools Intersect.

**Additional file 6: Table S5.** Annotation of the overlapping peaks using HOMER. Overlapping peaks were re-annotated using HOMERv4.7 with newly defined overlap peak lengths and table represents the chromosome-wise number of peaks associated with specific genomic regions like intergenic, intron, exon, 3′UTR, TTS, and promoter-TSS.

**Additional file 7: Table S6.** Repeat elements identification. Table represents the number of different types of repeat elements associated with HILS1.

**Additional file 8: Figure 3A.** Chr 1-16. Chromosome-wise distribution of rat linker histone HILS1. Each vertical line on the chromosomal map represents location of enriched regions as viewed in UCSC genome browser.

**Additional file 9: Figure 3B.** Chr 17-20 and ChrX. Chromosome-wise distribution of rat linker histone HILS1. Each vertical line on the chromosomal map represents location of enriched regions as viewed in UCSC genome browser.

**Additional file 10: Table S7.** LINE-1 subclass elements identification. Table represents the number of different types of subclasses of LINE-1 repeat elements associated with HILS1 and percentage of HILS1 occupancy to each subclass with respect to the total number of each in the rat genome.

**Additional file 11: Table S8.** Motif identification by MEME. Table represents the details of the most significant motifs identified from the overlapping peak summits (HILS1 ChIP peaks).

**Additional file 12: Table S1.** MACS 1.4.2 output file with annotations.A total of 32731 regions were selected as the overlapping peaks between the two replicates and the overlapping regions were annotated using HOMER.

**Additional file 13: Table S9.** PCR primer sequences used for ChIP-qPCR analysis.
