# Supplementary figures and images for: Spermatid-specific linker histone HILS1 is a poor condenser of DNA and chromatin and preferentially associates with LINE-1 elements
Source: Epigenetics Chromatin. 2018 Aug 1;11:43. doi: 10.1186/s13072-018-0214-0 (PMC6069787; doi:10.1186/s13072-018-0214-0)

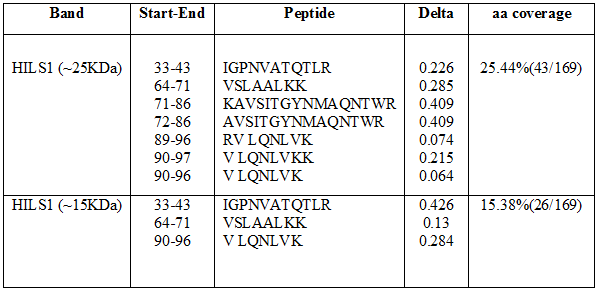

Supplement: Supplementary file 1 — Additional file 1: Figure S1. Mass spectrometric analysis of HILS1 IP bands confirms the specificity of HILS1 antibody. Specificity of the antibody raised against CTD of HILS1 was confirmed by the mass spec analysis of the immunoprecipitated bands. Results represent the peptides identified from 25 kDa and ~15kDa bands detected in western blot in both input and immunoprecipitated lane as represented in Figure 5A. Note that the prominent 25kDa band is the full-length form of HILS1, whereas ~15kDa showed different migration in HILS1 IP lane in comparison with input, which is the cleaved product. Coverage represents the percentage of sequence matching with peptides found in the analysis. [file 13072_2018_214_MOESM1_ESM.tif]

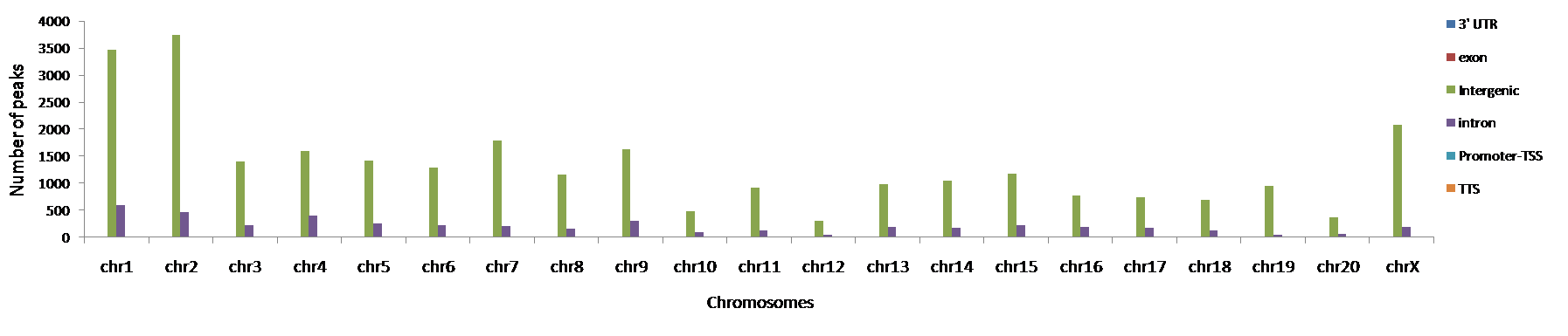

Supplement: Supplementary file 2 — Additional file 2: Figure S2. HILS1 mainly associates with intergenic regions across the chromosomes. ChIP sequencing was performed using anti-HILS1 antibodies and analysis of enriched genomic regions was carried out using MACS software. Figure represents the number of peaks associated with different genic elements (y-axis) distributed across different rat chromosomes (x-axis). Most of the peaks are associated with intergenic regions followed by introns across the chromosomes, whereas very less peaks are found in 3′UTR, exon or TSS regions. [file 13072_2018_214_MOESM2_ESM.tif]

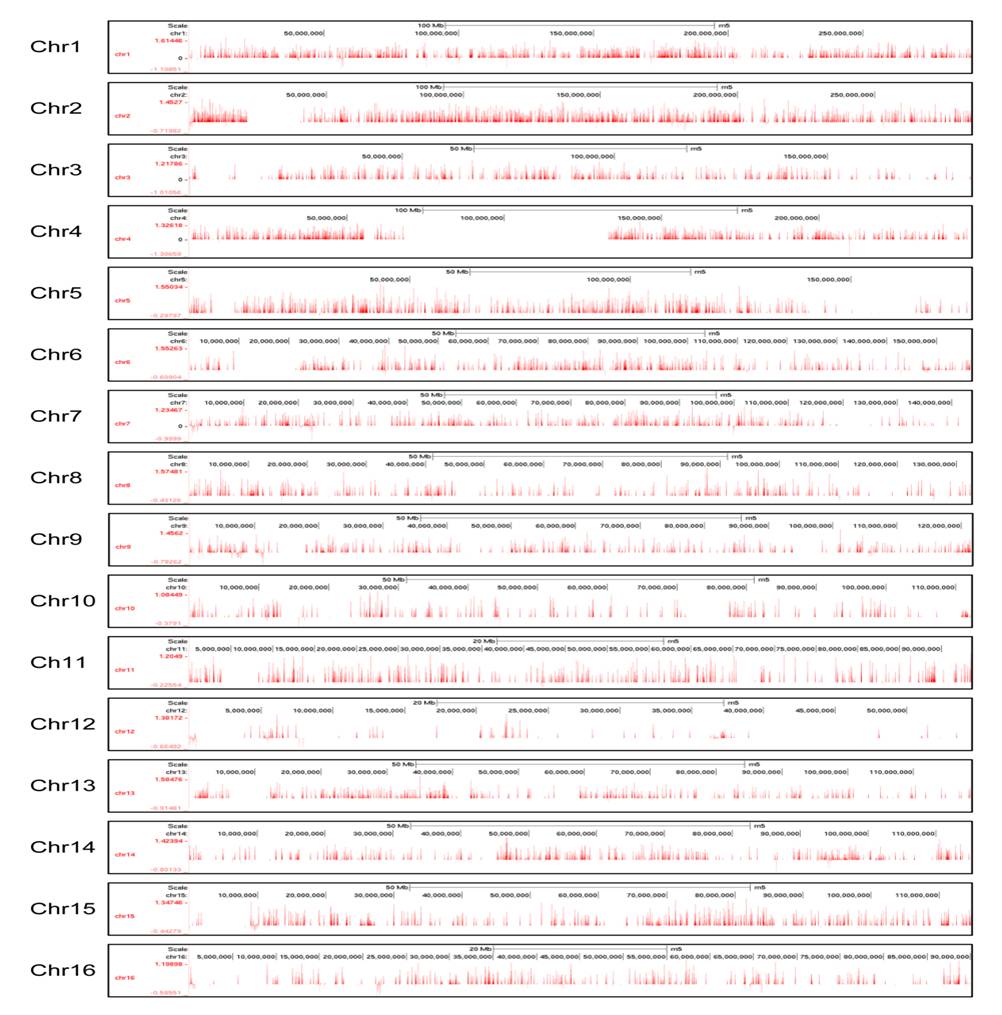

Supplement: Supplementary file 8 — Additional file 8: Figure 3A. Chr 1-16. Chromosome-wise distribution of rat linker histone HILS1. Each vertical line on the chromosomal map represents location of enriched regions as viewed in UCSC genome browser. [file 13072_2018_214_MOESM8_ESM.jpg]

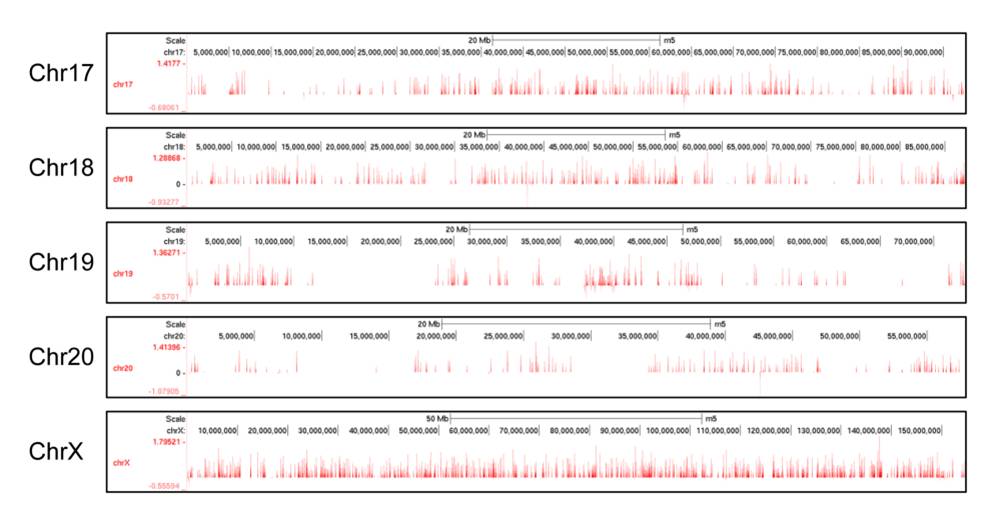

Supplement: Supplementary file 9 — Additional file 9: Figure 3B. Chr 17-20 and ChrX. Chromosome-wise distribution of rat linker histone HILS1. Each vertical line on the chromosomal map represents location of enriched regions as viewed in UCSC genome browser. [file 13072_2018_214_MOESM9_ESM.jpg]
